# Final analysis of the JALSG Ph+ALL202 study: tyrosine kinase inhibitor-combined chemotherapy for Ph+ALL

**DOI:** 10.1007/s00277-018-3323-8

**Published:** 2018-04-24

**Authors:** Yoshihiro Hatta, Shuichi Mizuta, Keitaro Matsuo, Shigeki Ohtake, Masako Iwanaga, Isamu Sugiura, Noriko Doki, Heiwa Kanamori, Yasunori Ueda, Chikamasa Yoshida, Nobuaki Dobashi, Tomoya Maeda, Toshiaki Yujiri, Fumihiko Monma, Yoshikazu Ito, Fumihiko Hayakawa, Jin Takeuchi, Hitoshi Kiyoi, Yasushi Miyazaki, Tomoki Naoe

**Affiliations:** 1 Division of Hematology & Rheumatology, Nihon University School of Medicine, 30-1 Oyaguchi, Itabashi-ku, Tokyo, 173-8617 Japan; 2 Department of Internal Medicine, Toyohashi Medical Center, Toyohashi, Japan; 3 Division of Molecular Medicine, Aichi Cancer Center Research Institute, Nagoya, Japan; 4 Department of Clinical Laboratory Science, Kanazawa University Graduate School of Medical Science, Kanazawa, Japan; 5 Department of Frontier Life Science, Nagasaki University Graduate School of Biomedical Sciences, Nagasaki, Japan; 6 Division of Hematology and Oncology, Toyohashi Municipal Hospital, Toyohashi, Japan; 7 Department of Hematology, Tokyo Metropolitan Cancer and Infectious Diseases Center, Komagome Hospital, Tokyo, Japan; 8 Department of Hematology, Kanagawa Cancer Center, Yokohama, Japan; 9 Department of Hematology/Oncology, Kurashiki Central Hospital, Kurashiki, Japan; 10 Department of Hematology, National Hospital Organization Minami-Okayama Medical Center, Hayashima, Japan; 11 Division of Clinical Oncology and Hematology, Department of Internal Medicine, Jikei University School of Medicine, Tokyo, Japan; 12 Department of Hemato-Oncology, Saitama International Medical Center, Saitama Medical University, Saitama, Japan; 13 Third Department of Internal Medicine, Yamaguchi University School of Medicine, Ube, Japan; 14 Department of Hematology and Oncology, Mie University Graduate School of Medicine, Tsu, Japan; 15 Department of Hematology, Tokyo Medical University, Tokyo, Japan; 16 Department of Hematology and Oncology, Nagoya University Graduate School of Medicine, Nagoya, Japan; 17 Dpartment of Hematology, Meirikai Chuo Genral Hospital, Tokyo, Japan; 18 Department of Hematology, Atomic Bomb Disease Institute, Nagasaki University, Nagasaki, Japan; 19 National Hospital Organization Nagoya Medical Center, Nagoya, Japan

**Keywords:** Imatinib, Allogeneic hematopoietic stem cell transplantation, Philadelphia chromosome-positive acute lymphoblastic leukemia

## Abstract

The Japan Adult Leukemia Study Group (JALSG) Ph+ALL202 study reported a high complete remission (CR) rate for Philadelphia chromosome-positive acute lymphoblastic leukemia (Ph+ALL) patients treated with imatinib-combined chemotherapy. However, the long-term treatment efficacy remains uncertain. Here, we report a final analysis of the JALSG Ph+ALL202 study. The outcomes were compared with those of the JALSG ALL93 and ALL97 studies, which were conducted in the pre-imatinib era. Ninety-nine newly diagnosed Ph+ALL patients were enrolled in Ph+ALL202 (median age, 45 years; median follow-up, 4.5 years). CR was achieved in 96/99 (97%) patients. Fifty-nine of these 96 patients (61%) underwent allogeneic hematopoietic stem cell transplantation (allo-HSCT) in their first CR (CR1). The 5-year overall and disease-free survival (DFS) rates were 50 and 43%, respectively, which were significantly higher compared to those in the pre-imatinib era (15 and 19%, respectively). Multivariate analysis revealed that imatinib administration, allo-HSCT in CR1, and a white blood cell count < 30 × 10^9^/L were favorable independent prognostic factors for long-term DFS. Improved odds of receiving allo-HSCT and a lower relapse rate leaded to good long-term outcomes. The 3-year DFS tended to be higher in PCR-negative than that in PCR-positive patients (29 vs. 14%) in the non-HSCT patients, and this tendency was also seen in the allo-HSCT patients (59 vs. 50%). The higher rate of CR upon imatinib use may have contributed to these improvements.

## Introduction

Imatinib, a potent inhibitor of the *BCR-ABL1* tyrosine kinase, possesses strong anti-leukemic activities for Philadelphia chromosome-positive acute lymphoblastic leukemia (Ph+ALL). In adult ALL, the Ph chromosome is the most frequent cytogenetic abnormality, identified in approximately 30% of patients [1]. Before the introduction of imatinib, the prognosis of Ph+ALL was poor, except in patients treated with allogeneic hematopoietic stem cell transplantation (allo-HSCT). At this time, although the complete remission (CR) was 60–70%, the relapse rate was high if allo-HSCT was not performed [2, 3].

In 2002, the Japan Adult Leukemia Study Group (JALSG) studied imatinib-combined chemotherapy for newly diagnosed Ph+ALL patients (the Ph+ALL202 study) and found a high CR rate for the initial 80 patients [4, 5]. Although several groups have reported similar results [6–11], the long-term prognosis of patients receiving imatinib-combined chemotherapy remains unclear and is still a major concern.

Herein, we report the final analysis of the end-of-study results of the Ph+ALL202 study for the enrolled entire 100 patients. The clinical outcomes were compared with those of the JALSG ALL93 and ALL97 studies, which were prospective studies in the pre-imatinib era, as a means to investigate the survival benefits of imatinib for Ph+ALL patients.

## Patients and methods

### Treatments

Ph+ALL patients were recruited from three major leukemia studies in Japan (JALSG ALL93, ALL97, and Ph+ALL202). The design of the JALSG Ph+ALL202 study, which investigated the effects of imatinib combined with chemotherapeutic agents, has been previously described in detail [4, 5]. Allo-HSCT was recommended if a human leukocyte antigen-identical sibling donor was available. A human leukocyte antigen-matched unrelated donor or cord blood transplantation within a two-locus mismatch was used at each institution’s discretion. Similarly, the treatment protocols of JALSG ALL93 and ALL97 have been described previously [12, 13]. All protocols were approved by JALSG and subsequently reviewed by the institutional review board of each participating center. All patients provided written informed consent before participation.

### Quantitation of *BCR-ABL1* transcripts

In the imatinib cohort, 367 bone marrow samples collected at days 28 and 63 of the induction course, and after the first, second, fifth, and sixth consolidation courses, were analyzed for *BCR-ABL1* transcripts by quantitative reverse transcriptase-polymerase chain reaction (PCR), as previously reported [5]. The threshold for quantification was 50 copies/μg RNA, corresponding to a minimal sensitivity of 10^−5^. Values below this threshold were categorized as PCR negativity. The minimal residual disease (MRD) at the time of allo-HSCT was evaluated by quantitative-PCR within 30 days prior to transplantation.

### Statistical analysis

Differences in overall survival (OS) and disease-free survival (DFS) probabilities between groups were estimated using Kaplan-Meier curves and the log-rank test [14]. OS was calculated from the time of the study entry to the time of death or last follow-up. DFS was measured from the time of achievement of CR until relapse or death from any cause. To investigate the impact of allo-HSCT as a time-varying covariate (TVC), the method presented by Gooley et al. was used [15]. TVC analysis was computed from the date of CR. The Andersen-Gill model was applied for the calculation of statistical significance in TVC [16]. Risk factors were evaluated by univariate and multivariate analyses (MVA). Demographic differences among the groups were evaluated using the chi-square or Wilcoxon rank-sum test, as appropriate. All statistical analyses were performed using STATA 12 software (STATA Corp., College Station, TX, USA).

## Results

### Patient characteristics

**Fig. 1:**
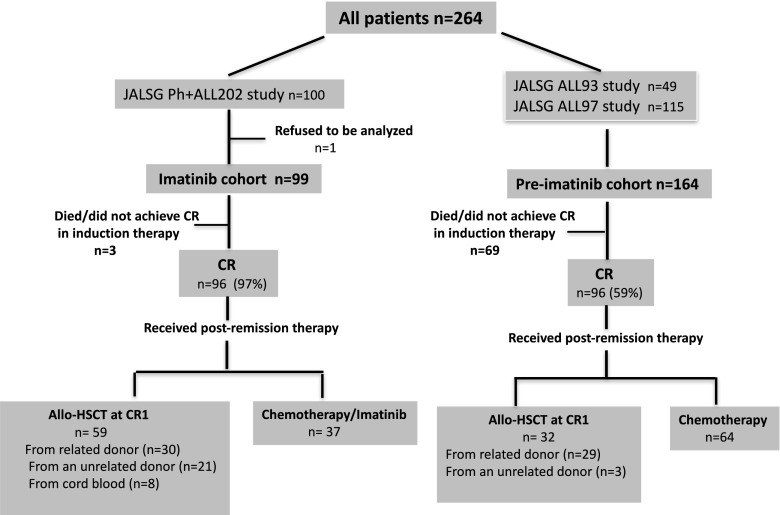
CONSORT diagram of all patients entered into the JALSG study. CR, complete remission; allo-HSCT, allogeneic hematopoietic stem cell transplantation

Figure 1 details the study flow. From December 1993 to February 1997, 263 patients were enrolled in JALSG ALL93, with 43 patients diagnosed with Ph+ALL. Between May 1997 and December 2001, 404 patients were enrolled in JALSG ALL97, with 121 patients diagnosed with Ph+ALL. Thus, 164 patients with Ph+ALL were enrolled before the approval of imatinib by the Japanese government; this group was analyzed as the pre-imatinib cohort in the present study. The median follow-up in the pre-imatinib cohort was 4.9 years (range, 0.4–8.0 years). One hundred patients were enrolled in Ph+ALL202 between August 2002 and May 2005 (imatinib cohort). Because one patient withdrew, 99 patients were finally included in the analysis. The median follow-up period in this cohort was 4.5 years (range, 0–9.1 years). The median white blood cell (WBC) count at diagnosis was 23.1 × 10^9^/L (range, 1.7–814.2). The minor *BCR-ABL1* transcript was expressed in 74 patients, with the major *BCR-ABL1* transcript expressed in the remaining 25. The median number of *BCR-ABL1* transcript copies at diagnosis was 3.7 × 10^5^ copies/μg RNA (range, 0.0091–72.0).

The patient characteristics of the pre-imatinib and imatinib cohorts are listed in Table 1. The age, sex, performance status (PS), additional chromosome status, WBC count (≥ 30 × 10^9^/L vs. < 30 × 10^9^/L), and positivity of cluster of differentiation (CD)13 and/or CD33 were similar between the pre-imatinib and imatinib groups. However, allo-HSCT at CR1 was more frequently performed in the imatinib era (32 [20%] vs. 59 [60%] patients; *P* < 0.001).

**Table 1:** Characteristics of the Ph+ALL patients (*n* = 263)

Characteristic	Pre-imatinib cohort (*n* = 164)	Imatinib cohort (*n* = 99)	*P*
Age (years)			
Median (range)	46 (15–63)	45 (15–64)	0.232
< 45	63 (38)	41 (41)	
45–54	69 (42)	32 (32)	
≥ 55	32 (20)	26 (26)	
Sex (male/female)	92/72	54/45	0.898
PS			
0	80 (49)	52 (53)	
1	61 (37)	36 (36)	
2–3	20 (12)	10 (10)	
Unknown	3 (2)	1 (1)	0.897
WBC at diagnosis			
< 30 × 10^9^/L	81 (49)	54 (55)	
≥ 30 × 10^9^/L	83 (51)	45 (45)	0.447
CD13 and/or CD33+			
Negative	41 (25)	38 (38)	
Positive	71 (43)	53 (55)	
Unknown	52 (32)	8 (8)	0.150
Cytogenetics			
t(9;22) only	83 (51)	45 (45)	
Other abnormalities	81 (49)	54 (55)	0.447
Achieved CR			
Yes	95 (58)	96 (97)	
No	69 (42)	3 (3)	< 0.001
Allo-HSCT in CR1			
Yes	32 (20)	59 (60)	
No	132 (80)	40 (40)	< 0.001

Unless otherwise specified, the data are presented as *n* (%)

*Ph+ALL*, Philadelphia chromosome-positive acute lymphoblastic leukemia; *PS*, performance status; *WBC*, white blood cell; *CD*, cluster of differentiation; *CR*, complete remission; *allo-HSCT*, allogeneic hematopoietic stem cell transplantation

In the pre-imatinib cohort, 12 patients relapsed after the approval of imatinib by the Japanese government (beyond December 2001). However, information on how many of these patients subsequently received imatinib-based therapy is lacking.

As the role of imatinib maintenance has not been well understood, imatinib maintenance was not allowed after completion of the protocol in the imatinib cohort. However, three patients used imatinib as maintenance therapy after allo-HSCT and none of them relapsed.

### Response

Figure 1 shows the patient flow and treatment outcomes. In the imatinib cohort, 96/99 (97%) patients achieved CR after a median of 29 days (range, 20–74 days) and after only one induction course. Two patients died during induction therapy: one of pneumonia and one of pulmonary bleeding. Another patient who discontinued imatinib because of ileus did not achieve CR. The overall CR rate was significantly higher in the imatinib cohort than that in the pre-imatinib cohort (95/164 patients, 59%; *P* < 0.001).

### Allo-HSCT

Allo-HSCT was performed in 32/95 (34%) CR1 patients in the pre-imatinib cohort. Most were from related donors. In the imatinib cohort, allo-HSCT was performed in 59/96 CR patients (61%) at CR1 from a related donor (*n* = 30), unrelated donor (*n* = 21), and single cord blood (*n* = 8). Fifty-two of 70 CR patients < 55 years and 7/26 patients ≥ 55 years underwent allo-HSCT in CR1. Nineteen patients < 55 years were not performed allo-HSCT at CR1. The reasons for not receiving transplantation were early relapse (12 patients), chemotherapy-related early death (2 patients), and unknown reason (4 patients). We have previously described the details of allo-HSCT in this study [17, 18]. The median times from CR1 to allo-HSCT were 132 (range, 56–522) and 124 days (range, 12–961) in the pre-imatinib and imatinib cohorts, respectively.

### Remission duration and survival

Overall, 57/99 patients died in the imatinib cohort. The major causes of death were transplant-related mortality (*n* = 29) and leukemia (*n* = 19). Other causes included chemotherapy-related causes (*n* = 4), pneumonia (*n* = 2), suicide (*n* = 1), malignant melanoma (*n* = 1), and lung cancer (*n* = 1).

**Fig. 2:**
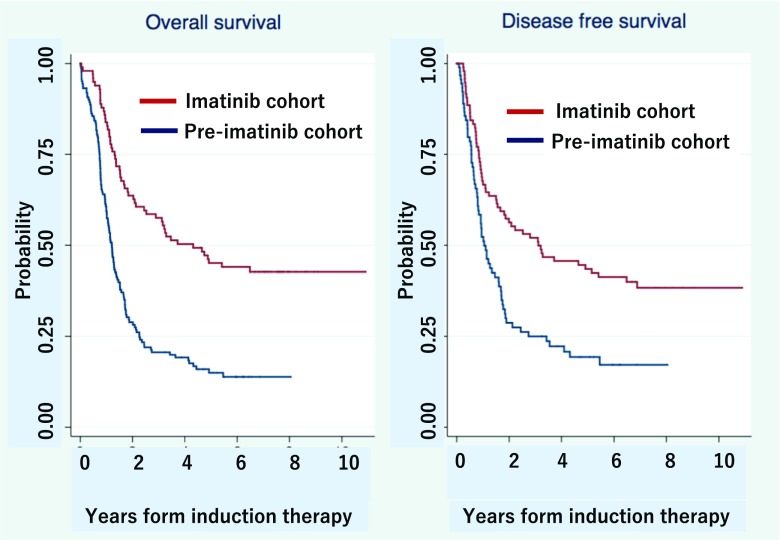
Survival curves of Ph+ALL202. Overall survival (OS) and disease-free survival (DFS) in the imatinib and pre-imatinib cohorts

The 5-year OS and DFS rates in the imatinib-cohort were 50% (95% confidence interval [CI], 40–60%) and 43% (95% CI, 33–53%), respectively; these were significantly higher compared to those in the pre-imatinib cohort (OS 15% [95% CI, 10–21%]; *P* < 0.001; and DFS 19% [95% CI, 11–29%]; *P* = 0.001) (Fig. 2 and Table 2). The imatinib cohort, allo-HSCT, and a WBC count < 30 × 10^9^/L were favorable factors for OS and DFS in both the univariate analysis and MVA (Table 3).

**Table 2:** Outcomes at 5 years according to the treatment received

	OS	P*	DFS	*P* ^*^
Total cases				
Imatinib cohort	50% (95% CI, 40–60%)	< 0.001	43% (95% CI, 33–53%)	0.001
Pre-imatinib cohort	15% (95% CI, 10–21%)		19% (95% CI, 11–29%)	
Patients receiving allo-HSCT in CR1				
Imatinib cohort	57% (95% CI, 43–69%)	0.229	55% (95% CI, 41–67%)	0.123
Pre-imatinib cohort	47% (95% CI, 28–63%)		40% (95% CI, 22–56%)	
Patients not receiving allo-HSCT in CR1				
Imatinib cohort	28% (95% CI, 15–42%)	0.025	26% (95% CI, 14–41%)	0.085
Pre-imatinib cohort	8% (95% CI, 4–13%)		8% (95% CI, 3–18%)	

*OS*, overall survival; *DFS*, disease-free survival; *CI*, confidence interval; *allo-HSCT*, allogeneic hematopoietic stem cell transplantation; *CR1*, first complete remission

*Log-rank test

**Table 3:** Results of the univariate and multivariate analyses among the total 263 patients

Variable	OS				DFS			
	Univariate analysis		Multivariate analysis		Univariate analysis		Multivariate analysis	
	RR (95% CI)	*P*	RR (95% CI)	*P*	RR (95% CI)	*P*	RR (95% CI)	*P*
Imatinib use before allo-HSCT								
No	1 (reference)		1 (reference)		1 (reference)		1 (reference)	
Yes	0.39 (0.29–0.54)	< 0.001	0.52 (0.37–0.74)	< 0.001	0.51 (0.36–0.74)	< 0.001	0.64 (0.44–0.94)	0.023
Age (years)								
< 45	1 (reference)		1 (reference)		1 (reference)		1 (reference)	
45–54	1.87 (1.33–2.61)	< 0.001	1.47 (1.05–2.08)	0.026	1.27 (0.84–1.92)	0.254	0.61 (0.38–0.98)	0.041
≥ 55	1.48 (1.01–2.17)	0.046	0.94 (0.58–1.54)	0.168	1.17 (0.75–1.81)	0.481	0.52 (0.31–0.88)	0.015
Sex								
Male	1 (reference)		NA		1 (reference)		NA	
Female	0.91 (0.68–1.22)	0.547			0.77 (0.54–1.10)	0.147		
PS								
0–1	1 (reference)		NA		1 (reference)		NA	
2–3	1.41 (0.92–2.17)	0.115			1.41 (0.83–2.38)	0.204		
WBC at diagnosis								
< 30 × 10^9^/L	1 (reference)		1 (reference)		1 (reference)		1 (reference)	
≥ 30 × 10^9^/L	1.72 (1.29–2.31)	< 0.001	1.74 (1.29–2.35)	< 0.001	1.50 (1.05–2.13)	0.024	1.8 (1.28–2.66)	0.001
Cytogenetics								
t(9;22) only	1 (reference)		NA		1 (reference)		NA	
Other abnormalities	1.06 (0.71–1.58)	0.761			1.08 (0.99–1.18)	0.088		
Allo-HSCT in CR1								
No	1 (reference)		1 (reference)		1 (reference)		1 (reference)	
Yes	0.30 (0.21–0.42)	< 0.001	0.41 (0.28–0.60)	< 0.001	0.32 (0.22–0.46)	< 0.001	0.24 (0.15–0.38)	< 0.001

*OS*, overall survival; *DFS*, disease-free survival; *HR*, hazard ratio; *CI*, confidence interval; *NA*, not available; *allo-HSCT*, allogeneic hematopoietic stem cell transplantation; *CR1*, first complete remission

### Imatinib impact in the allo-HSCT group

In the allo-HSCT group, the 5-year OS and DFS rates tended to be higher in the imatinib-cohort than those in the pre-imatinib cohort (57% [95% CI, 43–69%] vs. 47% [95% CI, 28–63%]; *P* = 0.229; and 55% [95% CI, 41–67%] vs. 40% [95% CI, 22–56%]; *P* = 0.123, respectively; Table 2), although the differences were not significant. In the MVA, imatinib administration did not significantly affect either the OS (hazard ratio [HR], 0.59; 95% CI, 0.31–1.11; *P* = 0.10) or the DFS (HR, 0.58; 95% CI, 0.32–1.06; *P* = 0.077) (Table 4). Cumulative incidence of relapse (CIR) at 5 years in the imatinib cohort was 15%. CIR of the pre-imatinib cohort, 23%, was not different from that of the imatinib cohort (*P = 0.40*).

**Table 4:** Cox regression analyses of the effects of imatinib on the clinical outcome

	Pre-imatinib cohort vs. imatinib cohort	
	Univariate analysis	Multivariate analysis
All patients		
OS	HR, 0.39; 95% CI, 0.29–054; *P < 0.001*	HR, 0.52; 95% CI, 0.37–0.74; *P < 0.001*
DFS	HR, 0.51; 95% CI, 0.36–0.74; *P < 0.001*	HR, 0.64; 95% CI, 0.44–0.94; *P = 0.023*
DFS*		HR, 0.53; 95% CI, 0.37–0.77; *P = 0.001*
Patients receiving allo-HSCT in CR1		
OS	HR, 0.68; 95% CI, 0.37–1.28; *P = 0.232*	HR, 0.59; 95% CI, 0.31–1.11; *P = 0.10*
DFS	HR, 0.63; 95% CI, 0.35–1.14; *P = 0.127*	HR, 0.58; 95% CI, 0.32–1.06; *P = 0.077*
Patients not receiving allo-HSCT in CR1		
OS	HR, 0.50; 95% CI, 0.33–0.76; *P = 0.001*	HR, 0.50; 95% CI, 0.30–0.85; *P = 0.010*
DFS	HR, 0.67; 95% CI, 0.42–106; *P = 0.087*	HR, 0.64; 95% CI, 0.39–1.07; *P = 0.088*

*OS*, overall survival; *DFS*, disease-free survival; *HR*, hazard ratio; *CI*, confidence interval; *allo-HSCT*, allogeneic hematopoietic stem cell transplantation; *CR1*, first complete remission

P < 0.05 was considered significant

*Time-dependent analyses considering hematopoietic stem cell transplantation in CR1 as a time-dependent event (Andersen-Gill model)

### Imatinib impact in the non-HSCT group

In the non-HSCT group, the 5-year OS in the imatinib cohort was significantly higher than that in the pre-imatinib cohort (28% [95% CI, 15–42%] vs. 8% [95% CI, 4–13%]; *P* = 0.025; Table 2). Imatinib administration remained a significant predictor of OS in the MVA (HR, 0.50; 95% CI, 0.30–0.85; *P* = 0.010; Table 4). Among non-HSCT patients, 44/63 (70%) and 28/37 (76%) patients in the pre-imatinib and imatinib cohorts, respectively, relapsed. The 5-year DFS in the imatinib cohort tended to be higher than that in the pre-imatinib cohort (26% [95% CI, 14–41%] vs. 8% [95% CI, 3–18%]; *P* = 0.085; Table 2). MVA revealed no significant impact of imatinib on DFS (HR, 0.64; 95% CI, 0.39–1.07; *P* = 0.088; Table 4).

### Clinical impact of allo-HSCT in the pre- and post-imatinib eras

**Fig. 3:**
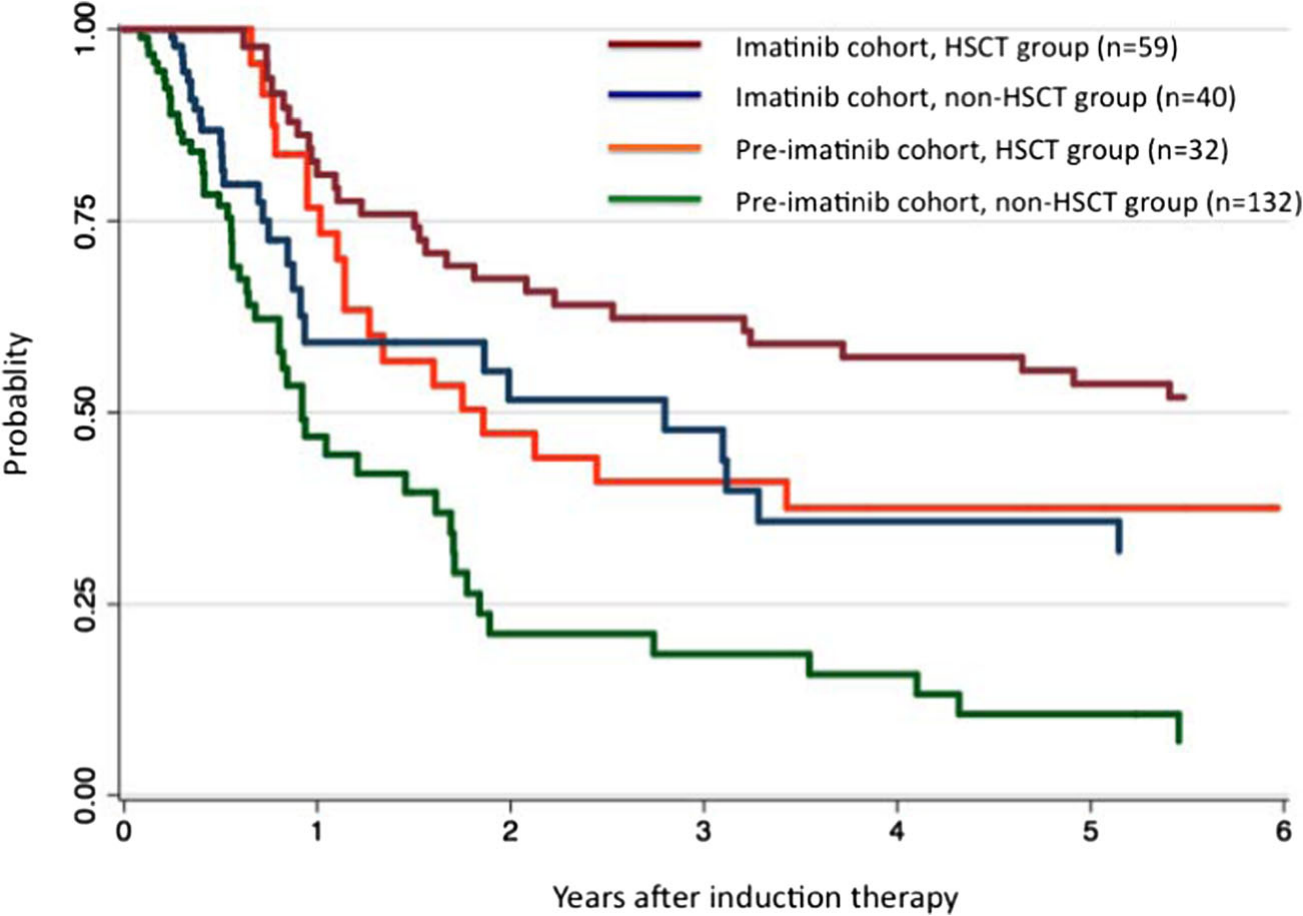
Disease-free survival by time-varying covariate analysis in the four treatment groups (pre-imatinib cohort, non-allogeneic hematopoietic stem cell transplantation [allo-HSCT] in first complete remission [CR1]; pre-imatinib cohort, allo-HSCT in CR1; imatinib cohort, non allo-HSCT in CR1; and imatinib cohort, allo-HSCT in CR1)

Direct comparison of patients receiving vs. not receiving transplantation seemed statistically invalid, since, in order to receive allo-HSCT, the patients must first survive for a sufficient period. Therefore, to compare the clinical impact of allo-HSCT between the pre- and post-imatinib eras, the survival probabilities with respect to allo-HSCT were assessed using the TVC method. Figure 3 shows a Kaplan-Meier plot of DFS by the TVC method. In the pre-imatinib cohort, the 5-year DFS in the allo-HSCT group (38% [95% CI, 21–54%]) was significantly higher than that in the non-HSCT group (11% [95% CI, 3–22%]) (*P* = 0.031). Conversely, in the imatinib cohort, there was no significant difference in the 5-year DFS between the allo-HSCT (54% [95% CI, 40–66%]) and non-HSCT groups (36% [95% CI, 19–53%]) (*P* = 0.133). MVA based on the Andersen-Gill model, including a time-dependent post-remission strategy, revealed that imatinib (HR, 0.52; 95% CI, 0.36–0.75; *P* = 0.001), allo-HSCT in CR1 (HR, 0.54; 95% CI, 0.35–0.84; *P* = 0.007), and low WBC count (HR, 1.75; 95% CI, 1.21–2.51; *P* = 0.003) were independent favorable prognostic factors for DFS.

Even in the imatinib era, the relapse rate was significantly higher in patients not undergoing (28/37 patients; 76%) vs. those undergoing allo-HSCT at CR1 (9/59 patients; 15%) (*P* < 0.05). The prognosis of relapsed patients was extremely poor, with only 4/28 relapsed patients not undergoing allo-HSCT in CR1 survival. For patients aged ≥ 55 years, none of the transplanted patients relapsed, although 2 patients in CR died of ileus and cerebral hemorrhage, 944 and 1202 days after allo-HSCT, respectively.

The median times from CR1 to allo-HSCT in the imatinib cohort were 124 days. We compared CIR for transplanted patients within 124 days from CR1 (early transplantation) and those after 124 days (late transplantation). CIR of early-transplanted patients was 14% which was not statistically different from that of the late-transplanted patients, 18% (*P = 0.72*).

Additional chromosomal abnormalities affected neither OS nor DFS. Complex, monosomal, monosomy 7, and double Ph+ karyotypes were seen in 27, 22, 15, and 2 patients in the imatinib cohort, respectively. In a subgroup analysis, relapse rate was not different in patients with and without complex karyotype (27 vs. 13% in allo-HSCT at CR1 patients, 58 vs. 70% in non-HSCT patients, respectively), with and without monosomal karyotype (8 vs. 19% in allo-HSCT at CR1 patients, 72 vs. 65% in non-HSCT patients, respectively), and with and without monosomy 7 (0 vs. 13% in allo-HSCT at CR1 patients, 71 vs. 64% in non-HSCT patients, respectively).

### Toxicity

The toxicity observed in Ph+ALL202 was similar to that seen with conventional chemotherapy. Although some patients required imatinib interruption, none withdrew from the study due to adverse events. In the induction phase, > 20% reduction of imatinib administration was observed in eight patients because of ileus (*n* = 3), grade 3 nausea (*n* = 2), grade 3 hepatic damage (*n* = 1), and grade 4 myelosuppression (*n* = 2). After achieving CR, no unacceptable adverse events were noted.

### MRD analysis in the imatinib cohort

In 93/99 patients in whom PCR assay results were available during the early phase of therapy (post-induction or after first consolidation therapy) in the imatinib cohort, 67 patients (72%) were PCR negative. Of these, 39 patients underwent allo-HSCT in their CR1. The 3-year DFS tended to be higher in PCR-negative than that in PCR-positive patients (29% [95% CI, 14–46%] vs. 14% [95% CI, 7–46%]; *P* = 0.329) in the non-HSCT group, and this tendency was also seen in the allo-HSCT cohort (59% [95% CI, 41–73%] vs. 50% [95% CI, 26–70%]; *P* = 0.380). In the pre-imatinib cohort, MRD assays were not available and were hence not included in the present analysis.

## Discussion

Before the introduction of imatinib, the prognosis of Ph+ALL was quite poor. JALSG reported that the CR rates of Ph+ALL were 64% (ALL87) [19], 58% (ALL90) [20], 51% (ALL93) [12], and 56% (ALL97) [13]; these were inferior to those of Ph-negative ALL. Similar results have been reported from Western countries [21, 22].

However, after the introduction of imatinib, the prognosis of Ph+ALL has dramatically improved. We previously reported excellent results of the JALSG Ph+ALL202 study (CR rate, 96%; event-free survival, 60%; median follow-up, 1 year) [4, 5]. Similarly, several other groups have demonstrated relapse-free survival rates of approximately 50% at 1.5–2 years [7, 8]. However, the long-term outcomes of Ph+ALL patients treated with imatinib and chemotherapy remain unclear and have recently attracted attention. In this report, we demonstrated the long-term outcome of the JALSG Ph+ALL202 study by, during a long observation period, comparing the outcomes of newly diagnosed Ph+ALL patients prospectively treated in the imatinib or pre-imatinib eras.

In the imatinib cohort, although 8% of our patients required imatinib dose reduction in the induction phase because of grade 3/4 adverse events, the regimen was generally well tolerated and feasible. Furthermore, this regimen was better tolerated after CR, with the adverse events being similar to those of chemotherapy alone. Moreover, the OS and DFS were significantly higher compared to those in the pre-imatinib cohort, and in the MVA, imatinib administration showed significant favorable effects on OS and DFS.

We previously reported that imatinib-based therapy improved the OS rate after allo-HSCT [17, 23]. Recently, several other studies also reported survival benefits of imatinib-based therapy. The NILG09/00 study reported that imatinib-treated patients showed significantly better OS and DFS compared to those in the historical group (38 vs. 23% and 39 vs. 25%, respectively) [24]. In that study, imatinib also contributed to improvements of DFS in patients not receiving allo-HSCT (8 vs. 0%), despite relatively few patients being analyzed (imatinib-treated group, *n* = 15; chemotherapy alone, *n* = 13). More recently, Fielding et al. reported the clinical outcomes of the UKALLXII/ECOG2993 study. The 4-year OS and DFS rates of the imatinib cohort were significantly greater compared to those in the pre-imatinib cohort (38 vs. 22% and 33 vs. 18%, respectively). MVA taking allo-HSCT into account showed a modest additional benefit of imatinib for DFS, but not for OS. Thus, the authors concluded that imatinib facilitated allo-HSCT and improved survival [25].

The OS in the Ph+ALL202 study was higher than that given in the above reports. The 5-year OS rate was 50%, as compared to 38% in UKALLXII/ECOG2993 at 4 years and NILG09/00 at 5 years. Furthermore, in the UKALLXII/ECOG2993 study, 46% of imatinib-treated patients underwent allo-HSCT [24], which was lower than that observed in the Ph+ALL202 study. Interestingly, the total imatinib dose in NILG09/00 was lower than that in the Ph+ALL202 study [24], indicating that the use of a sufficient dose of imatinib along with allo-HSCT contributed to the good outcome in our study. The OS rate in the present study was almost similar to that of a Korean study, in which imatinib (600 mg) was continuously administered [26]. Moreover, the recent report from the GRAALL study group suggested that imatinib may be more important than intense chemotherapy may be [27].

In Ph+ALL202, 59/96 (61%) CR patients underwent allo-HSCT in CR1. Relapse occurred in only 9/59 (15%) patients, as compared to in 28/37 (76%) patients not undergoing allo-HSCT in CR1. The 5-year DFS rate was superior for the transplanted group (55% vs. non-transplanted, 26%), although it was not statistically significant. Recently, Daver et al. reported that addition of allo-HSCT provided no benefit for chemotherapy (hyper-CVAD) + imatinib-treated patients. However, because of the small number of patients undergoing allo-HSCT in their study, this finding may be limited [28]. In our previous report, the short-term survival of imatinib-combined chemotherapy alone was comparable with that followed by allo-HSCT [5]. However, long-term analysis identified late relapse as a major concern for patients not receiving allo-HSCT, with the latest relapse occurring 3.1 years after CR, and the prognosis of relapsed patients was extremely poor. Herein, only 4/28 relapsed patients not undergoing allo-HSCT in CR1 survived. Therefore, early allo-HSCT likely provides survival advantages after imatinib-combined chemotherapy. For elderly patients, several studies have reported that the CR rate of imatinib-combined chemotherapy was approximately 90%, but with a median survival duration of only 20–23.5 months [29–31]. Ravandi et al. reported that patients aged > 40 years showed a poor survival rate after allo-HSCT [32]. In our series, 24% of patients ≥ 55 years underwent allo-HSCT, none of whom relapsed, suggesting that allo-HSCT should be the treatment of choice even for elderly patients.

As the duration of the consolidation period before allo-HSCT has not been established in Ph+ALL, in our imatinib cohort, relapse rate between early-transplanted (within 124 days from achieving CR1) and late-transplanted patients (after 124 days from achieving CR1) was not different. Early transplantation may be recommended to avoid early relapse or chemotherapy-related complications.

In the pre-imatinib era, MRC UKALLXII/ECOG2993 demonstrated a median OS of 13 months in Ph+ALL patients. However, only 28% of patients actually underwent allo-HSCT, as designed by their protocol. The authors reported that the majority of CR patients could not receive allo-HSCT because of age limitations or early events even when a donor was available [33]. Conversely, in our imatinib cohort, almost 60% of CR1 patients underwent allo-HSCT, indicating that imatinib-combined chemotherapy induces a high remission rate and durable response, improving the odds of allo-HSCT. However, in our imatinib cohort, it should be concerned that 12 patients (< 55 years) could not receive allo-HSCT in CR1 because of early relapse. Intensification with next-generation TKI may decrease early relapse.

Before the introduction of imatinib, the prospective LALA-94 trial demonstrated a 3-year survival rate of 37% in the transplantation group, despite 95% of patients receiving allo-HSCT [34]. Herein, we found that allo-HSCT after imatinib-combined chemotherapy tended to be associated with improved OS and DFS compared with that before the imatinib era. The minimal residual status at allo-HSCT also has clinical impact on the relapse risk [35]; 78% of patients attained molecular CR by PCR with our regimen, which probably resulted in the better outcome compared with that in the pre-imatinib era.

A notable point is that in Ph+ALL202, imatinib was administered from days 8 to 63 together with other anti-cancer agents and also for 28 days as consolidation therapy. A synergistic or additive activity of imatinib with other anti-leukemic agents has been suggested [36], and several investigators have reported good response rates of imatinib and chemotherapy combination as frontline therapy for Ph+ALL [6–9]. Hence, we consider that a sufficient dose and duration of imatinib, as well as concomitant use with other anti-cancer agents, can provide significant survival benefits for Ph+ALL patients. Additionally, recent study has shown that autologous HSCT after imatinib therapy is also beneficial [37]. Thus, the clinical relevance of autologous HSCT in patients with Ph+ALL should be investigated as an alternative stem cell source in the tyrosine kinase inhibitor era.

The only unfavorable prognostic factor in our study was a WBC count > 30 × 10^9^/L, and this is in agreement with previous JALSG studies [12, 13, 20]. Although mutational analysis for *BCR-ABL1* was not performed in our study, the presence of various *BCR-ABL1* mutations, such as T315I, may have affected the relapse rate [38]. Recently, several studies reported the prognostic significance of additional cytogenetic abnormalities in imatinib era. PETHEMA group identified prognostic impact of monosomal karyotype [39]. Monosomy 7 is another risk factor in some reports. Deletion of *IKF1* residing on chromosome 7 possibly contributes to poor prognosis in Ph+ALL. In our study, additional cytogenetic abnormalities including monosomal karyotype and monosomy 7 did not affect outcome. Future studies will help clarify the unfavorable characteristics and precise indication of allo-HSCT for Ph+ALL.

To properly interpret our current results, the strengths and limitations must be considered. A major strength is the large sample size from consecutive prospective clinical trials in Japan, which provides a better estimation of the endpoints and adds statistical power to the analyses. On the other hand, one possible limitation is the presence of both known and unknown residual confounding factors. First, we did not perform an exhaustive analysis of the relationship between clinical outcome and MRD in the pre-imatinib cohort. Recently, Yoon et al. reported that in the TKI-treated patients, poor molecular responders were high risk for relapse [40]. Despite the lack of comparative data of MRD in the pre-imatinib cohort, 72% of the evaluable patients were PCR-negative during the early phase of therapy in the imatinib cohort. We believe that the powerful anti-leukemia activity of the imatinib-based therapy mostly contributed to the prevention of subsequent relapse in the present analysis. We do not know how long imatinib maintenance should be continued. Patients with continuous molecular remission for 2 or 3 years possibly able to stop imatinib administration as has been reported in chronic myelogenous leukemia (CML). Second, there may have been differences in the chemotherapy regimens used. However, there were no significant differences in OS and DFS between JALSG ALL93 and ALL97 for Ph+ALL patients (data not shown), and the chemotherapeutic regimen in JALSG Ph+ALL202 was similar to those used in these protocols. Thus, the effectiveness of chemotherapeutic agents can be considered similar between the cohorts. Third, changes in the transplantation procedures throughout the study period (1993–2005) should also be considered. In Japan, the widespread use of alternative donors after 2000 facilitated the extension of allo-HSCT eligibility, and this might have boosted the high frequency of allo-HSCT in the imatinib cohort.

Taken together, imatinib-combined chemotherapy appears effective and feasible in the long term. This regimen offered the patients an improved chance of allo-HSCT, which resulted in excellent therapeutic outcomes. Moreover, imatinib-based therapy provided survival benefits for Ph+ALL patients not receiving allo-HSCT. In the tyrosine kinase inhibitor era, molecular assays for prognostic genes, in addition to MRD monitoring, allow us to identify patients who would benefit from treatment intensification and to select the subsequent therapy in patients not undergoing allo-HSCT.
